# Lessons from aviation safety: “plan your operation – and operate your plan!”

**DOI:** 10.1186/s13037-014-0038-1

**Published:** 2014-09-27

**Authors:** Steven R Schelkun

**Affiliations:** Surgical Consultant, Naval Medical Center, San Diego, USA

The systematic implementation of surgical safety checklists in the past 5 to 10 years has contributed to a global decrease in morbidity and mortality after surgical procedures [1-5]. What I find intriguing about these data is that the significant reduction in adverse events with the utilization of a simple surgical check list did not require a costly, high-tech solution but rather a simple, almost “no-cost attitude” and procedural change in the operating theater. Going back in history, in response to adverse aircraft incidents, the Army-Air Corps test pilots developed a series of aircraft check-lists to reduce the work load on the pilot of the complex aircraft systems and instrumentation in 1935. The aviation community has adopted these check lists and procedures as standard not only in military, but commercial and civilian aviation procedures as well. Their premier goal has always been the safety of passengers and aircraft.

Aviation flight planning consists of four distinct steps:

1. Plan the flight using aviation charts to determine how to get from point A to point B.
2. File a flight plan with the FAA providing pertinent details of the aircraft, altitude of flight, route, aircraft fuel capacity and endurance and time enroute. This step also includes defining an alternate plan (i.e. alternate airport) in case the airplane cannot safely land at the intended destination.
3. A detailed pre-flight equipment and instrument check of the aircraft itself, engine and navigation instruments, and fuel, oil and aircraft systems to ensure that all systems are working properly.
4. Standardized communication with ground control, the tower, and constantly along the route of flight with Air Traffic Control.

This ‘4-step procedure’ in conjunction with use of the standardized checklists and mandatory ‘readbacks’ represent the basic tenet of proven aviation safety principles [6]. In flight training, a pilot is taught to “*Plan your flight* – *and fly your plan*!” As a result, the aviation community has boasted a safety record that is unparalleled in the military, civilian and commercial transportation world. Granted that there are aircraft accidents, but the vast majority can be traced to deviation of the standard procedures.

As an orthopaedic trauma surgeon, my colleagues and I routinely face urgent and emergency situations in patients with multiple injuries and complex medical problems. On a daily basis, we have to orchestrate in the operating room with a team of personnel dealing with an outrageous number of complex instrument sets and a huge inventory of different implants. It is easy to understand how errors of omission of one of the steps could potentially lead to adverse results and a higher incidence of morbidity and mortality. While the implementation of surgical safety checklists certainly had a global impact in reducing preventable adverse events, I strongly feel that checklists alone fall short of addressing the entire aspect of pre-operative planning. As surgeons, we not only desire improved patient safety, but we also strive for improved patient functional outcomes. Checklists can help with the mechanical procedures in the operating room, but we also need to address the quality of the operative procedure by developing a mindset and intent for a more successful operation. This process starts long before the patient gets to the operating room.

The aviation flight planning scenario is remarkably applicable to the surgical field.

Modeled on the aviation safety principle, I propose a similar ‘4-step procedure’ for a standardized pre-operative planning in surgery:

1. Plan your operation. First of all understand the patient, the injury, the goals of the operation and choose a surgical procedure to get you there. In orthopaedic trauma surgery this is where templating of the fracture with the surgical implants will help you better understand the fracture, understand the goals of reduction and alignment as well as determine the type, location and size of the implant that you need.
2. Develop a detailed surgical tactic by thinking through the operation chronologically, one step at a time. Decide what operating table, patient position, drape pack, prepping technique and surgical approach you will use. This is a good time to review the details of a less frequently used approach. Be prepared for obstacles along the way in the way of important anatomic structures and have an alternate “Plan B” to handle potential problems along the way.
3. Develop a surgical instrument checklist to let the operative team know what you need. Every fracture can have different nuances that may require a different instrument and implant set from the inventory of over 85 fracture sets in my hospital. I cannot expect the surgical team to know what I need nor pull the proper sets unless I tell them specifically. Pre-printed surgeon preference cards do not work well in trauma surgery due to the variability of our cases and the number of sets.
4. Communicate all along the way with your operative team. The team is there to help you achieve your goals with the patient. They are professionals as well and want to be part of a successful team. By discussion with them the patient’s problem, your goals and intended plan of approach you are building teamwork for success. When you give them your equipment and instrument list before the case you are allowing them to prepare with you for the case as well. When the patient is in the room, positioned on the table and the entire team is assembled you can then call for a “Time Out” [7]. Each member of the team has a specific task. I normally stand at the foot of the bed with my check list and address the items one at a time, requiring a verbal reply, the “readback” in aviation terminology, for each item on the list [6].

Each member should be given the opportunity to ask questions or express any concerns about the patient’s condition, the plans or completeness of the preparations. I was brought up to believe that the operating surgeon was the “Captain of the Ship” and what he or she said was the rule. I no longer feel that is a sustainable paradigm in the operating room. Better yet, I like to think of the surgeon as the leader of a professional team, who is willing to listen to the other members of the team with mutual respect. It is this respect and open communication that will allow a member of the team to speak up if he observes something that he thinks may impact or contribute to the success of the operation. A standardized checklist usually takes no more than 30–45 seconds but reaps enormous benefits in the confidence the team has in you and your case [4,8].

As a formal ‘debriefing’ at the end of each case, I place the post-operative x-rays on the view box (or DICOM equivalent for comparison) next to the pre-op x-rays and my pre-operative planning template and compare my results with the plan. If they don’t match, I need to know why.

I then ask myself and my team two questions:

1. “*What went well with this operation*?” (to reinforce successful planning)
2. “*What could I do differently next time to improve the results*?” (to close the loop in the learning process in each case)

This structured debriefing approach validates good decisions and allows us to learn what could be done better next time. Pre-operative planning is much more than signing the site and reading through a few checklists so we don’t forget to give the antibiotics before the case. Pre-operative planning is a mind-set for success of the operation and a process that if done conscientiously will not only make you a better surgeon, build a better surgical team, but will improve your surgical results and improve the safety for your patient.

As surgeons and educators we are obligated to teach our residents and fellows not only how to operate, but we need to help them develop a mind-set and process that will improve the surgical results and keep our patient safe in the future.

Therefore, in every case, it is imperative for a surgeon to “*Plan your operation* – *and operate your plan*!”.
